# Microbial diversity and community structure of denitrifying biological filters operated with different carbon sources

**DOI:** 10.1186/s40064-016-3451-3

**Published:** 2016-10-07

**Authors:** Yingxue Sun, Dandan Shen, Xiaoli Zhou, Na Shi, Yuan Tian

**Affiliations:** 1 Department of Environmental Science and Engineering, Beijing Technology and Business University, No. 11 Fucheng Road, HaiDian District, Beijing, 100048 People’s Republic of China; 2 State Environmental Protection Key Laboratory of Microorganism Application and Risk Control (SMARC), School of Environment, Tsinghua University, Beijing, 100084 People’s Republic of China

**Keywords:** Wastewater tertiary treatment, Denitrifying biological filter, Biofilm, Microbial community, Carbon source

## Abstract

The treatment performance and spatial microbial community structure of three parallel denitrifying biological filters (DNBFs) operated with methanol, ethanol and acetate, respectively, were explored. The acetate-fed DNBF presented the highest denitrification rate and NO_x_-N (NO_2_-N and NO_3_-N) removal efficiency, while the methanol-fed DNBF showed the lowest carbon consumption and NO_x_-N removal efficiency. Distinct spatial distribution patterns of terminal restriction fragment length polymorphism fingerprints were observed among the DNBFs. The ethanol enhanced captured biofilms throughout the flowpath of DNBF had the highest diversity and evenness. The methanol-enhanced attached biofilm along the flowpath presented the highest evenness, but lowest richness and limited diversity. *β*-*Proteobacteria* was dominant in the microbial community in all of methanol, ethanol and acetate enhanced biofilm; however, different external carbon sources resulted in different dominant genera species. *Thauera was* dominant in the acetate enhanced bacterial community, while both *Dechloromonas* and *Thauera* were dominant in that of ethanol fed. However, methylotrophic bacteria (*Methyloversatilis* and *Methylotenera*) dominated exclusively in the methanol enhanced bacterial community throughout of the DNBF.

**Electronic supplementary material:**

The online version of this article (doi:10.1186/s40064-016-3451-3) contains supplementary material, which is available to authorized users.

## Background

Conventional secondary-treated municipal wastewater usually contains appreciable amounts of oxidized nitrogen and other nutrients, which pose a risk of eutrophication to receiving waters. Also, elevated nitrate concentrations were proved to have both lethal and non-lethal effects on a number of commercially relevant aquatic species (Hamlin et al. 2008). Denitrifying biological filter (DNBF) is extensively considered as an effective, economical, stable and feasible technology to control oxidized nitrogen from secondary effluents of municipal wastewater treatment plants (Jeong et al. 2006). A DNBF performs the denitrifying function through biological conversions of organics and oxidized nitrogen in absence of oxygen by the biofilms attached on granular media, meanwhile achieves a physical removal of suspended particles by the media filtration. Denitrifying bacteria in the biofilm play an important role in transforming nitrate to nitrogen gas, while organic carbon as the denitrifying electron donor is a significant factor to perform a complete denitrification process.

The organic matters in secondary effluents are commonly low to meet the demands of electron donors for anoxic denitrification and energy for cell growth and maintenance (Hallin et al. 2006). Hence, external organic carbon is required for wastewater tertiary denitrification process to avoid incomplete denitrification and nitrite accumulation. The external organic carbon always includes common organic carbon source (e.g. methanol, acetate and ethanol) and alternative carbon source (e.g. hydrolysis products of primary sludge and solid waste, glycerin-based byproduct of biofuel production) (Cherchi et al. 2009; Lu et al. 2014). Under a given plant size, hydraulic load, influent water quality and operation conditions of a denitrification process, the types of external organic carbons pose significant impacts on the external carbon dosage, nitrogen removal efficiency, denitrifying rates and bio-kinetics (Hallin et al. 1996; Hallin and Pell 1998; Cherchi et al. 2009; Rocher et al. 2015). Such effects could mainly be attributed to that different electron donors (external carbon source) lead to different denitrifying microbial ecosystems (Guven 2009; Lu et al. 2014). In addition, carbon types have influence on the expression levels of carbon oxidases (e.g., alcohol dehydrogenase catalyzing methanol and glycerol oxidation) (Baytshtok et al. 2009; Lu et al. 2011).

The use of molecular techniques has contributed to the determination of the exogenous carbon source as one of the controlling factors determining the structure and function of the denitrifying microbial community, during anoxic denitrification (Kraft et al. 2011; Warneke et al. 2011; Lu et al. 2014). Using stable-isotope probing, full-cycle rRNA analysis, and fluorescence in situ hybridization-microautoradiography (FISH-MAR), Ginige et al. (2004; 2005) characterized methanol-fed and acetate-fed denitrifying microbial community in sequencing batch reactors, respectively, and found *Methylophilales* bacteria were the dominant denitrifiers in methanol-fed denitrifying sequencing batch reactor while *Comamonadaceae* and *Rhodocyclaceae* were the dominant denitrifiers in the acetate-fed reactor. Osaka et al. (2008) characterized the differences of microbial community structure between two active sludge reactors using acetate and methanol as the external carbon source by using terminal restriction fragment length polymorphism (T-RFLP) and cloning analysis. Baytshtok et al. (2009) demonstrated that *Methyloversatilis* and *Hyphomicrobium* were dominant methylotrophic bacteria in a denitrifying sequencing batch reactor and the concentration of *Hyphomicrobium* decreased significantly when switching the electron donor from methanol to ethanol by stable isotope probing ^13^C 16S rRNA gene clone libraries and real-time quantitative polymerase chain reaction assays. In addition, effects of different alternative carbon sources on denitrifying microbial community structure were also carried out using polymerase chain reaction (PCR) based molecular techniques or high-throughput techniques (Warneke et al. 2011; Lv et al. 2014).

Molecular techniques obviously bring us valuable information on microbial community of wastewater denitrification; however, most studies about the impacts of different carbon sources on denitrifying microbial ecosystem were focused on suspended active sludge systems. Moreover, since bioflm-based reactors usually enriches more diverse communities than that of active sludge system (Lu et al. 2011, 2014), there are still considerable gaps in the knowledge of biofilm-based systems. Recently, the study carried out by Srinandan et al. (2012) investigated the effects of different exogenous carbon sources (acetate, glucose, methanol and ethanol) on denitrifying biofilm structure by denaturing gradient gel electrophoresis and FISH, and found that nitrate removal efficiency was low in ethanol-fed biofilm but the denitrifying bacteria abundance was high. However, this study focused on the biolfim attaching on polystyrene slides, which were suspended in an active sludge reactor; related investigation on biofilm in denitrifying filter system is limited.

In this study, spatial microbial community diversity and structure for three parallel DNBFs operated with common used external organic carbon of methanol, ethanol and acetate were investigated. The following specific objectives were pursued based on the DNBFs achieving satisfactory nitrogen removal: a. evaluating the treatment performance of the DNBFs influenced by different external carbon source; b. determining the spatial variations and diversity of community composition and dominant species of the three DNBFs by DNA fingerprinting T-RFLP technique combined clone library; c. making a insight on the microbial community structure of DNBF reactors impacted by methanol, ethanol and acetate.

## Methods

### Experimental reactor description

**Fig. 1:**
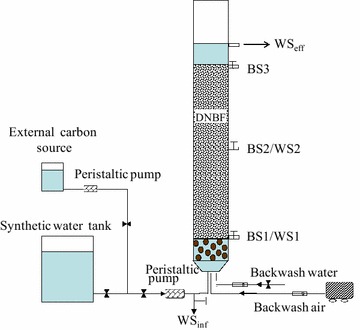
Schematic diagram of a lab-scaled DNBF system. *WS*_*inf*_, *WS1*, *WS2* and *WS*_*eff*_ are water-sampling points. BS1, BS2 and BS3 are biofilm sampling points

Three paralleling lab-scaled up-flowed DNBFs fed by acetate (R1), ethanol (R2) and methanol (R3), respectively were set up. Each DNBF was made of plexiglass column with a height of 600 mm and a diameter of 80 mm (working volume of 2.5 L) (Fig. 1). The packed height of filter material (frosted globosely glass beads with uniform diameter of 4 mm, specific surface area of 5.58 cm^2^/g and bulk density of 2.7 × 10^3^ kg/m^3^) was 400 mm. In the bottom of DNBF, there was a gravel layer with a height of 50 mm to support the filtering layer. The influent of synthetic wastewater and the external carbon source were mixed in the pipe before pumped to the bottom of the DNBF reactor by a peristaltic pump, which also control the filtration velocity. The DNBF was backwashed every 5 d for 15 min with combined air and water. During backwashing, the water flow rate was 7 L/(m^2^ s) and the airflow rate was 15 L/(m^2^ s).

Three sampling ports (BS1, BS2 and BS3) were constructed for filter materials sampling at 0, 200 and 400 mm from the bottom of the filter layer. Also, three taps (WS1, WS2 and WS_eff_) were equipped for liquid sampling at 0, 200 and 450 mm from the bottom of the filter layer. The inlet and outlet water samples for the DNBF were WS_inf_ and WS_eff_ as shown in Fig. 1.

### DNBF reactor operation

The DNBF reactors were inoculated with activated sludge taken from one anoxic tank of a sewage treatment plant in Beijing, China with anaerobic/anoxic/aerobic (A^2^/O) system to enhance biofilm forming on the beads. The DNBFs were fed with synthetic wastewater, which prepared by tap water added with carbon source (acetate, ethanol and methanol, respectively), NO_3_-N (KNO_3_), phosphate buffer and mineral medium with trace elements (including H_3_BO_3_ 2.86 g/L, ZnSO_4_·7H_2_O 0.22 g/L, CuSO_4_·5H_2_O 0.08 g/L, MnSO_4_·4H_2_O 2.03 g/L and Na_2_MoO_4_·2H_2_O 1.26 g/L, which added as 0.1 mL/L to the synthetic wastewater). At the start-up stage, each DNBF reactor was operated at a hydraulic loading of 14–19 m^3^/(m^2^ day) and with chemical oxygen demand (COD) and nitrate nitrogen (NO_3_-N) of the inlet (WS_inf_) were 490–570 and 48–65 mg/L, respectively. Each DNBF reached a steady state after operation for 8 weeks at room temperature (20–25 °C).

Under steady state, each DNBF was operated with a hydraulic loading of 29 m^3^/(m^2^ day). The concentration of COD and NO_3_-N of the inlet (WS_inf_) were 230–380 and 25–38 mg/L, respectively. The high level of COD in the influent was to ensure sufficient carbon source for denitrifying bacteria. Each DNBF was continually running for 2 months for this experiment, in which COD, NO_3_-N, nitrite nitrogen (NO_2_-N), pH and DO were monitored every 2 days of the water samples (WS_inf_, WS1, WS2 and WS_eff_) to examine the dynamics of nutrient removal efficiency. Concentrations of COD, NO_3_-N and NO_2_-N were measured according to standard methods (APHA 1998). The level of pH and DO was determined by a pH sensor (pHS-25) and a DO sensor (WTW Oxi 340i), respectively.

### Biofilm samples collection and DNA extraction

The biofilm was classified to two forms: captured biofilm (CB) and attached biofilm (AB) (Jeong et al. 2006). The beads with biofilm were sampled from the sampling ports (BS1, BS2 and BS3) of each DNBF (R1, R2 and R3), and then firstly washed using milliQ water until there was no obvious adhesion on them, and then the washed liquid was collected as CB suspension. While the biofilm retained on the beads were put into a centrifuge tube with milliQ water (45 mL) and shaked by a vortex mixer at 3000 rpm for 5 min, and then the detached biomass was decanted from the centrifuge tube and collected as AB suspension (Delatolla et al. 2008). All the CB and AB suspensions were diluted with milliQ water to 100 mL and stored at 4 °C for the subsequent extraction of total DNA.

Total DNA was extracted using the sodium dodecyl sulphate (SDS)-cetyl trimethyl ammonium bromide (CTAB)-based DNA extraction method (Douterelo et al. 2013). Each biofilm suspension sample was put into a centrifuge tube and centrifuged at 12,000 r/min (4 °C) for 5 min, and then the deposition was mixed with Tris-EDTA (567 µL) and blended until resuspension. Thereafter, 30 μL SDS (10 %) and 10 μL proteinase K were added and mixed and then incubated at 37 °C for 1 h. 100 μL NaCl (5 mol/L) and 80 μL CTAB/NaCl were sequential added mixed and incubated at 65 °C for 10 min. After blended with equal volumes of phenol–chloroform–isoamyl alcohol mixture (25:24:1), the sample was then centrifuged at 12,000 r/min (4 °C) for 10 min. The supernatant were decanted to a 2 mL centrifuge tube and mixed with 0.8-fold volumes of isopropyl alcohol, then centrifuged at 12,000 r/min (4 °C) for 5 min. The deposition was rinsed with 1 mL alcohol (70 %) and centrifuged at 12,000 r/min (4 °C) for 10 min, and then the DNA was pelletised. Followed by the air dry, the extracted DNA was resuspended in 95 μL Tris-EDTA buffers (pH 8.0) and stored at −20 °C.

### PCR amplification of the 16S rRNA gene

The extracted DNA was amplified by polymerase chain reaction (PCR) using a TC-512 analyzer (TECHNE, Bibby Scientific, UK). For clone library construction and sequencing, 16S rRNA gene from 1 µL DNA extract was PCR-amplified using specific primers 27F (5′-AGA GTT TGA TCC TGG CTC AG-3′) and 1492R (5′-GGT TAC CTT GTT ACG ACT T-3′) (Lane 1991). For T-RFLP analysis, 16S rRNA gene from 1 µL DNA extract was PCR-amplified using eubacterial universal primers 8F (5′-AGA GTT TGA TCC TTG GCT CAG-3′) and 1492R, and the forward primer 8F was fluorescently labeled at the 5′ end with 6-carboxyfluorescein (6-FAM) (Zhang et al. 2011). All PCR reactions with a final volume of 25 μL including 12.5 μL 2×Taq PCR colorless Mix (Dingguo Biotech, China), 1 μL of each forward and reverse primer, 1 μL DNA template and 9.5 μL dd H_2_O. The PCR reactions were operated under the following thermal profile: The PCR amplification parameters were as follows: 5 min initial denaturation at 95 °C and then 30 cycles for denaturing at 94 °C and 1 min, thereafter 1 min for annealing at 55 °C, and 1.5 min fro elongation at 72 °C, with 10 min for final extension at 72 °C and a hold at 4 °C. PCR products were verified the product size by electrophoresis on 1.5 % (w/v) agarose gels. PCR products were purified using DNA Fragment Quick Purification/Recover Kit (Dingguo Biotech, China).

### T-RFLP analysis

T-RFLP analysis of bacterial 16S rRNA gene was applied to analyze the denitrifier community of biofilm samples collected from DNBFs. The purified fluorescent PCR products (10 μL) was digested with 3 U of the restriction enzyme MspI (Thermo Scientific, USA) for 4 h at 37 °C, and then inactivated at 65 °C for 10 min (Zhang et al. 2011). The final reactions were submitted to a commercial company (Sunbioech Beijing, China) for sequencing using ABI 310 genetic analyzer with the GeneScan mode (Applied Biosystems, USA).

Peak Scanner software (Applied Biosystems/Life Technologies, Carlsbad, CA, USA) was used to analyze the T-RFLP fingerprints. The relative abundance of a terminal restriction fragment (T-RF) was evaluated by calculating the ratio of the peak area of a T-RF to the total peak area of all peaks within one sample. T-RFs that differed by smaller than 1 bp were clustered. Peaks with a relative abundance below 1 % were excluded from further analysis. Also, T-RF length smaller than 50 bp and larger than 900 bp were neglected to avoid uncertainties associated with fragment size determination.

Based on T-RFLP profiles, denitrifier structural diversity between attached and captured biofilm along the flowpath within a DNBF and the DNBFs operated by different carbon sources were evaluated by Shannon diversity index (*H*) and evenness (*E*) (Zhang et al. 2011). Shannon diversity index (*H*) of each sample was calculated by the equation of $H = \sum\nolimits_{i = 1}^{S} {\left( {p_{i} { \ln }p_{i} } \right)}$, where *p*_i_ is the ratio of individual RF peak relative intensity to the sum of the relative intestity of all RFs. Evenness (*E*) was calculated as $E = H/H_{max}$, where *H*_max_ is the maxium value of *H* and equal to ln*S*, and *S* was the sum of all peaks of each sample profile.

### Cloning and sequencing

The purified PCR fragments were ligated into a pGEM-T cloning vector (Promega, USA) and cloned into *Escherichia coli* according to the manufacturer’s instructions. Transformants were selected by using ampicillin resistance, while blue-white screening was employed to identify clones with inserts. The white colonies of ampicillin-resistant transformants were picked randomly and cultured overnight in LB broth containing 50 mg/L ampicillin. The randomly selected clones were conducted and sequenced by a commercial company (Dingguo Biotech, China). All the 16S rRNA gene sequences were subjected to a BLAST search engine at NCBI GenBank and identified through sequence similarities (http://blast.ncbi.nlm.nih.gov/Blast.cgi). Clones were sequenced and grouped based on a 95 % similarity criterion (Tindall et al. 2010). In total, three 16S rRNA gene clone libraries were constructed for the acetate, ethanol and methanol-fed DNBF, respectively.

### T-RFs identification and phylogenetic assignment

The observed T-RFs were identified by cloning and sequencing. The obtained bacterial clones from the 16S rRNA gene clone library of each DNBF were subjected to virtual T-RF simulations and examined by in silico enzymatic digestion with MspI (http://tools.neb.com/NEBcutter2/index.php). The virtual T-RF obtained from the in silico enzymatic digestion was then compared to the actual T-RF lengths obtained from the samples. A specific clone was considered present in the sample only if the virtual T-RFs matched the T-RFLP fingerprints of the biofilm sample (Lepère et al. 2006). The virtual T-RFs and the actual T-RFs were considered as the same when their length gap was less than 3 bp. Some clones with incomplete sequences at the region of the 8F forward primer were filled with a sequence from a close relative (González et al. 2000).

A T-RF length from the T-RFLP fingerprint was considered to be a single operational taxonomic unit (OTU) (Hallin et al. 2005). The clones from the 16S rRNA gene clone library of each DNBF can be divided into different OTUs on the basis of the virtual T-RFs. Moreover, some bacterial clones presenting different phylogenies but the same length of the virtual T-RFs were divided into sub- OTUs (such as OUT 1a and OUT 1b, which presented same T-RF length but different genera).

The MEGA software was used for alignment, calculation of the distance matrices for the aligned sequences and construction of neighbor-joining phylogenetic trees. Heat maps displaying relative abundance of specific bacteria taxonomies of the DNBFs were generated using R.

## Results and discussion

### Performance of the DNBF reactors

**Fig. 2:**
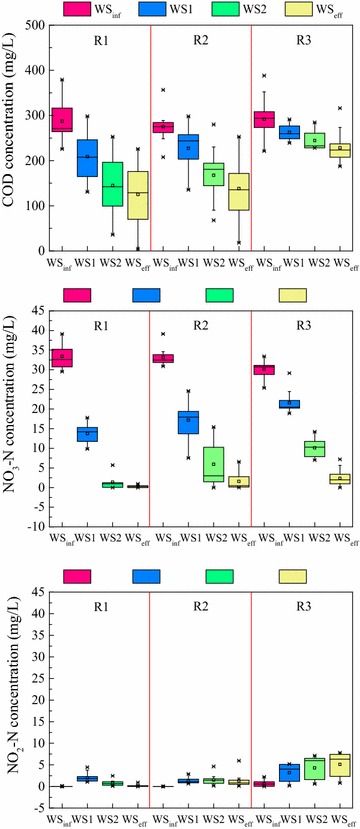
Concentrations of COD, NO_3_-N and NO_2_-N along the flowpath of each DNBF (n = 30). *R1*, *R2* and *R3* were acetate, ethanol and methanol-fed DNBF, respectively. The water samples of flowpath are in the sequence *WS*_*inf*_–*WS1*–*WS2*–*WS*_*eff*_

The spatial distribution of COD, NO_3_-N and NO_2_-N of acetate, ethanol and methanol-fed DNBF at steady operating state were shown in Fig. 2. The concentration of COD and NO_3_-N of each DNBF decreased along the flowpath under the non substrate-limited denitrifying growth conditions. The influent NO_3_-N of acetate, ethanol and methanol-fed DNBF were 33.1 ± 3.2, 33.6 ± 2.6 and 30.1 ± 1.8 mg/L, respectively, while the effluent NO_3_-N were 0.33 ± 0.19, 0.59 ± 0.73 and 2.28 ± 1.86 mg/L, respectively, indicating a 99, 98.3 and 92.4 % NO_3_-N removal of each DNBF. Moreover, the denitrificaton rate of acetate, ethanol and methanol-fed DNBF were 2.40, 2.29 and 2.01 kg NO_3_-N/m^3^ day, respectively. The acetate enhanced DNBF showed the highest denitrificaton rate and the NO_3_-N removal efficiency, which is in accordance with previous reports that acetate augmentation leading to a higher rate of denitrification than that of methanol and ethanol (Hallin et al. 1996).

Nitrite accumulation at the bottom (sampling port of WS1) of the acetate, ethanol and methanol-fed DNBFs was 17, 9 and 15 %, respectively. The NO_2_-N concentration decreased gradually along the flowpath of the acetate and ethanol-fed DNBFs, and the average nitrite at the outlet of the acetate and ethanol-fed DNBFs were 0.16 and 1.34 mg/L. However, a markedly nitrite accumulation occurred along flowpath of the methanol-fed DNBF, and the average nitrite up to 5.11 mg /L at the outlet of the reactor. The elevated level of nitrite along the flowpath of methanol-fed DNBF might decrease the denitrification rate or take further time to achieve complete removal of nitrite, for the reaction catalyzed by the nitrite reductase enzyme is considered as the limiting stage for anoxic denitrification process (Guven 2009).

The NO_x_-N (NO_2_-N and NO_3_-N) removal efficiency of acetate, ethanol and methanol-fed DNBF was 98.5, 94.2 and 75.9 %, respectively. Due to a relatively long adaptation period required for a methanol added microbial reactor (Hallin et al. 1996; Hallin and Pell 1998), the denitrification rate and NO_x_-N removal efficiency may be lower than that of DNBF fed by ethanol or acetate under the same start-up period and the steady operating state. Furthermore, different carbon metabolic routes and the involved enzymes may lead to different NO_x_-N removal efficiencies between methanol, ethanol and acetate-fed denitrifying bacteria. Acetate is directly converted to acetyl-CoA by the bacterial cell prior to entering the tricarboxylic acid cycle (TCA cycle), while ethanol is oxidized to acetaldehyde and subsequently to acetate and begin the biochemical pathways as well as that of acetate; however, methanol is initially utilized by bacterial cell in serine/glyoxylate pathways (Hallin and Pell 1998; Cherchi et al. 2009).

COD_consumed_ to NO_x_–N_reduced_ ratio of the acetate, ethanol and methanol-fed DNBF were 4.9, 4.3 and 2.9, respectively. Under the same operation conditions, the COD_consumed_ to NO_x_–N_reduced_ ratio of acetate and ethanol were higher than that of the theoretical stoichiometric ratio (denitrifyication consumption of a carbon source including the conversion of nitrate to nitrogen gas and microorganism growth), while the actual ratio of methanol was similar with that theoretical stoichiometric ratio. In this experiment, the synthetic wastewater input to the reactors was not deoxygenated, so the COD consumption of a carbon source primarily includes three parts as the conversion of nitrate to nitrogen gas, the removal of oxygen from the system and the production of extracellular material by other reactions (Hamlin et al. 2008). The average DO concentration at the effluent of acetate, ethanol and methanol-fed DNBF was 0.11, 0.13 and 0.36 mg/L, respectively under the same operating condition during, which indicated that acetate and ethanol-fed DNBFs might consume more external carbon for required denitrification and oxygen removal.

### Spatial distribution of microbial community and diversity of the DNBFs

**Fig. 3:**
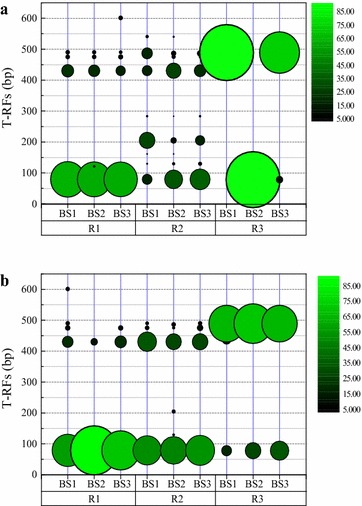
Distribution of T-RFs relative abundance of the biofilm samples along the flowpath of DNBF. **a** captured biofilm, **b** attached biofilm. *R1*, *R2* and *R3* were acetate, ethanol and methanol-fed DNBF. *BS1*, *BS2* and *BS3* were biofilm sampling at 0, 200 and 400 mm from the bottom of the filter layer

The microbial community and diversity of the DNBFs was analyzed using 16S rRNA gene T-RFLP fingerprinting (data in Additional File 1: Fig. S1). Figure 3 shows the spatial distribution of TFs relative abundance of the captured and attached biofilm samples from acetate, ethanol and methanol-fed DNBF, respectively. The T-RFs of 79, 121, 430, 475, 490 and 601 bp were observed in the acetate-fed DNBF, of which 79 and 430 bp were absolutely the dominant T-RFs for both captured and attached biofilms along the flowpath of DNBF. The ethanol-fed DNBF presented two dominant T-RFs (i.e. 79 and 430 bp) of the attached biofilm along the flowpath and the T-RFs of 79 bp showed the highest relative abundance, which is similar with that spatial distribution pattern of the attached biofilm of the acetate-fed DNBF. However, T-RFs spatial distribution of the ethanol enhanced captured biofilm remarkably displayed alternate dominant T-RFs between 79, 205, 430 and 486 bp along the flowpath.

Only three T-RFs of 78, 437 and 489 bp were observed in the methanol enhanced both attached and captured biofilm, which had no overlapped RFs with either acetate or ethanol-fed biofilm. Such phenomenon might be caused by the different metabolic properties between methanol fed bacteria and those fed by acetate and ethanol. The relative abundance of T-RFs at 489 bp of the methanol enhanced attached biofilm was relatively stable with around 65–71 % along the flowpath, while that of T-RFs at 78 bp increased from 18 to 34 % along the flowpath. However, alternate dominant T-RFs between 489 and 78 bp along the flowpath for the captured biofilm of methanol -fed. Comparing with the form of attached biofilm, captured biofilm was not so closely attached to the filter materials and was easily influenced by the water flowing state and intermediates produced during denitrification.

**Fig. 4:**
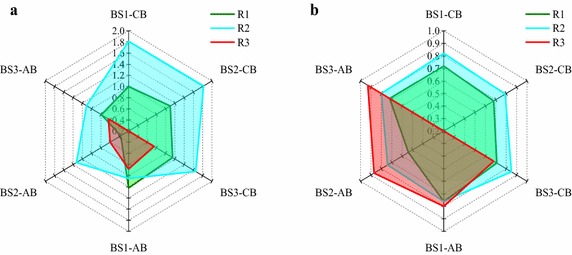
The diversity indices of biofilm samples from different external carbon source fed DNBFs. **a** Shannon diversity index (*H*); **b** Shannon evenness (*E*). *R1*, *R2* and *R3* were acetate, ethanol and methanol-fed DNBF. *BS1*, *BS2* and *BS3* were biofilm sampling at 0, 200 and 400 mm from the bottom of the filter layer. CB and AB were captured biofilm and attached biofilm

The microbial community diversity of all the biofilm samples fed by different carbon sources was evaluated by Shannon indices (Fig. 4). The ethanol augmented captured biofilm throughout the flowpath of DNBF presented the highest diversity and evenness, while that of methanolic augmentation showed the lowest. Such result complied with that the growth of denitrifying bacteria growth is most favored with ethanol augmentation because ethanol catabolism allowed formation of an energy source (NADH_2_) for the microorganisms (Gómez et al. 2000). The diversity index also revealed that ethanol and acetate enhanced captured biofilm presented similar distribution pattern along the flowpath of DNBF. However, for the attached biofilm, the sample in the bottom of the acetate-fed DNBF had the higher diversity index than the same site samples from the other DNBFs, which indicated that acetate was apt to be converted by denitrifying microorganism and to be promoted to a higher denitrification rate than the other external carbon source. Additionally, the methanol enhanced attached biofilm samples had the highest evenness and lowest richness among the DNBFs.

### Microbial community structure influenced by different carbon sources

Clone libraries of acetate, ethanol and methanol-fed DNBFs were constructed from the biofilm samples of R1-BS1-AB, R2-BS2-CB and R3-BS1-AB, respectively, which presented the highest Shannon diversity among all those samples within a DBPF. The obtained clones from 16S rRNA gene clone library of each DNBF were subjected to virtual T-RF simulations and were examined by in silico enzymatic digestion with MspI. Then the correlation of T-RFLP peaks and species were established by using in silico digestion. When comparing the virtual T-RFs with actual T-RFs, T-RFs of 87, 423, 489 and 511 bp emerged in acetate-fed DNBF, T-RFs of 80, 82 and 95 bp emerged in ethanol-fed DNBF and T-RFs of 192, 464 and 490 bp emerged in methanol-fed DNBF; however, some actual T-RFs disappeared in the virtual T-RFs. Thus, 25 OUTs were divided for denitrifying bacteria of all DNBFs based on T-RFLP profile combined clone libraries and silico enzymatic digestion (Additional File 1: Table S1).

**Fig. 5:**
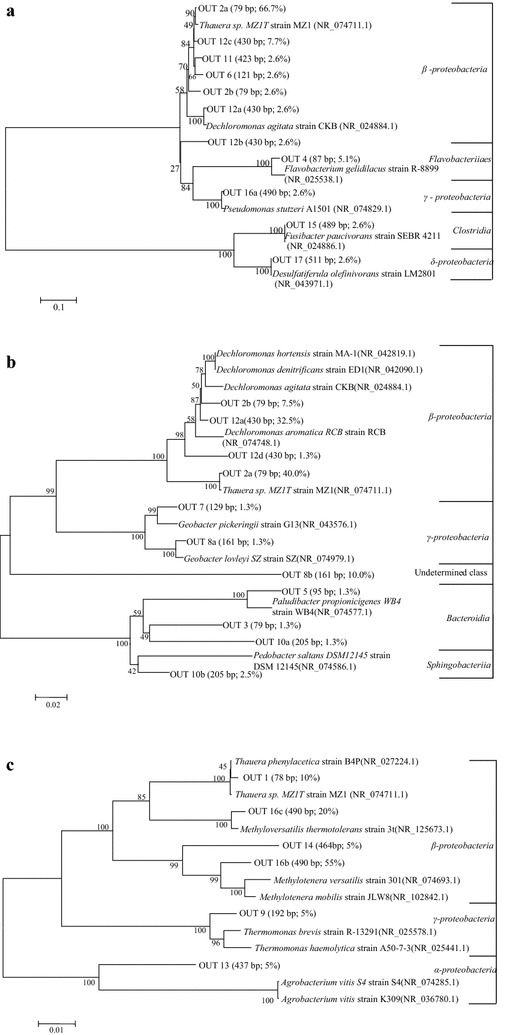
Phylogenetic trees of 16S rRNA gene sequences retrieved from different external carbon source fed biofilm clone library. **a** Acetate-fed; **b** ethanol-fed; **c** methanol-fed. The phylogenetic tree was constructed using a neighbor-joining algorithm with Jukes–Cantor distance in MEGA. The T-RFs length and abundance of each OTU in the clone library are shown in *parentheses*

From Venn diagram of the OTUs (Additional File 1: Fig. S2), the acetate and ethanol augmented biofilm shared the 3 same OTUs, however, that of methanol had exclusive 6 OTUs. Such result indicates that methanol-fed microbial community structure is considerably different from that of acetate and ethanol-fed. Also, there are some differences in the microbial community structure between acetate and ethanol-fed. The 16S rRNA gene sequences from the clones representing each of the OTUs were aligned with reference strains and are presented in the phylogenetic trees (Fig. 5).

Eight OTUs of the acetate-fed biofilm fell into three big phylogenetic groups of *Proteobacteria* (92 %, including *β*, *γ* and *δ*-*proteobacteria*), *Bacteroidetes* (5 %) *and Firmicutes* (3 %). The most dominant *β*-*proteobacteria* class, mainly including the genera *Thauera* (81 %, represented by OUT 2a, OUT 6, OUT 11 and OTU12c) and *Dechloromonas* (5 %, represented by OUT 2b and OTU12a) belonging to the family *Rhodocyclaceae*, accounted for 86 % of the total *Proteobacteria.* Additionally, other genera *Pseudomonas* (*γ*-*proteobacteria* class), *Desulfatiferula* (*δ*-*proteobacteria* class), *Flavobacterium* (*Flavobacteriia* class*, Bacteroidetes* phylum) and *Fusibacter* (*Clostridia* class*, Firmicutes* phylum) were identified for the acetate-fed biofilm. Eight OTUs of the ethanol-fed biofilm fell into two big phylogenetic groups of *Proteobacteria* (84 %, including *β* and *δ*-*proteobacteria*) and *Bacteroidetes* (6 %), while the class of 10 % clone sequence could not be determined. *β*-*proteobacteria* class was also the largest class among the denitrifying bacteria fed by ethanol with dominant genera of *Thauera* (41 %, represented by OUT 2a) and *Dechloromonas* (40 %, represented by OUT 2b, OTU12a and OTU12d). Furthermore, genera *Geobacter* (*δ*-*proteobacteria* class), *Paludibacter (Bacteroidia* class, *Bacteroidetes* phylum) and *Pedobacter (Sphingobacteriia* class, *Bacteroidetes* phylum) were identified for the ethanol-fed bacteria community. The dominated genus *Thauera* has been confirmed to be a typical denitrifier in wastewater denitrification enhanced by acetate or ethanol (Lu et al. 2014). *Dechloromonas* has been demonstrated as a major population in a field-scale ethanol enhanced denitrifying fluidized-bed reactor (Hwang et al. 2006), and was also dominant in this study and was only second to *Thauera.* Therefore, the dominant genera *of Thauera and Dechloromonas,* typical denitrifying bacteria, resulted in the high denitrification rate and NOx-N removal efficiencies of the acetate and ethanol-fed DNBFs.

Six OTUs of methanol enhanced bacteria were all belonged to *Proteobacteria* phylum with subdivision of *α, β,* and *γ*-*proteobacteria*, of which *β*-*proteobacteria* class was the largest group (90 %) including the genera *Thauera* (10 %, represented by OUT 1), *Methyloversatilis* (20 %, represented by OUT 16c) and *Methylotenera* (60 %, represented by OUT 14 and OUT 16b). *Methylotenera* and *Methyloversatilis* were exclusively found in the methanol - fed bacterial community among the 3 DNBFs. The dominant species *Methylotenera* and *Methyloversatilis* have been identified in various methanol enhanced denitrification systems and were further classified to obligate (growing on C1 compounds only) or restricted facultative (growing on C1 and multi-carbon compounds) methylotrophs (Mustakhimov et al. 2013; Lu et al. 2014). *Methyloversatilis* expresses a classic membrane-bound nitrate reductase and typical methylotrophy metabolic pathways during reducing nitrate to nitrite in anoxic conditions (Lu et al. 2012; Mustakhimov et al. 2013). *Methylotenera*, the most abundant genus in this methanol enhanced biofilm-based system, had also been proved to be one of the major species that consume methanol in situ (Kalyuhznaya et al. 2009). However, the denitrifying metabolic pathway of *Methylotenera* may be incomplete and will lead to accumulation of nitrous oxide for the lack of nitrous oxide reductase (Kalyuhznaya et al. 2009; Mustakhimov et al. 2013), which might result in the lowest denitrification rate and NOx-N removal efficiency of the methanol-fed reactor among the 3 DNBFs.

**Fig. 6:**
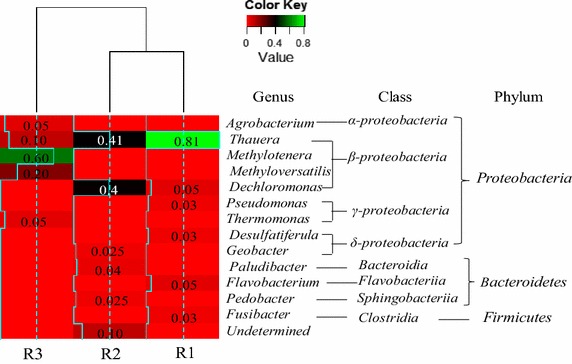
Heatmap of bacterial community structure of the DNBFs. *R1*, *R2* and *R3* were acetate, ethanol and methanol-fed DNBF

In general, the results showed that *Proteobacteria* phylum dominated the bacteria community of the DNBFs enhanced by the different carbon sources and followed by *Bacteroidetes* (Fig. 6), and such phenomenon is consistent with some previous reports (Lu et al. 2014). Also, *β*-*proteobacteria* class presented the largest group in all of the DNBFs. The acetate and ethanol-fed bacterial community shared the common dominant genus *Thauera*. Different from that of methanol-fed biofilm, genus *Thauera* was absolutely the largest species and accounted for 81 % of the acetate-fed biofilm clones. *Thauera* and *Dechloromonas* accounted for 41 and 40 %, respectively, of the acetate-fed biofilm clones. Furthermore, the methanol-fed biofilm exclusively occupied an amount of methylotrophic bacteria (*Methyloversatilis* and *Methylotenera*), which led the methanol enhanced DNBF to a limited diversity (Chistoserdova et al. 2009) and an initial long lag phase (Nyberg et al. 1992). Moreover, genra *Agrobacterium* and *Thermomonas* were uniquely observed in the methanol-fed biofilm. *Agrobacterium* belongs to the family *Rhizobiaceae* (*α*-*Proteobacteria* species), which was prevalent denitrifiers in some mountain ecosystems (Rich et al. 2003), while *Thermomonas* belongs to the family *Xanthomonadaceae* (*γ*-*proteobacteria* species) and had been reported in the community structure of denitrifying cathodic biofilms (Wrighton et al. 2010).

Spatial distribution of dominant denitrifying bacteria of the acetate, ethanol and methanol-fed DNBF, respectively, could be inferred by T-RFLP fingerprinting combined clone library. In terms of the attached biofilms, which exhibited immobility and stability comparing to captured bioflims, genera *Thauera* (related to T-RF of 79 bp) and *Dechloromonas* (related to T-RF of 430 bp) dominated the first and second abundance genera throughout the flowpath of both the acetate and ethanol -fed DNBF, while the relative abundances of *Thauera* along the flowpath of ethanol-fed were higher than that of acetate-fed. However, distinct spatial distribution patterns of dominant denitrifying bacteria of attached biofilm of ethanol-fed DNBF were observed, which *Methylotenera* and *Methyloversatilis* (related to same T-RF length of 490 bp) dominated the first abundance genera, while the relative abundance of the second dominated genus *Thauera* (related to T-RF of 78 bp) increased throughout the flowpath. At the inlet of methanol-fed DNBF, microorganisms growing on methanol use methylotrophy metabolic pathways (as serine/Glyoxylate Pathway), thereafter, some intermediates of denitrification and metabolism (as acetyl-CoA) might enter TCA cycle (Cherchi et al. 2009) along the flowpath from the bottom to top of the DNBF. Thus, genus *Thauera* increased along the flowpath of the methanol -fed DNBF.

## Conclusions

The acetate-fed DNBF presented the highest denitrification rate and NO_x_-N removal efficiency. Distinct spatial distribution patterns of T-RFLP fingerprints along the flowpath of the DNBFs were caused by the different external carbon sources. The ethanol enhanced captured biofilms throughout the flowpath of DNBF had the highest diversity and evenness, while that of methanol enhanced biofilms was the lowest. *β*-*proteobacteria* class presented the largest group in all acetate, ethanol and methanol-fed biofilm. *Thauera* and *Dechloromonas* dominated the acetate and ethanol enhanced denitrifiers, which might result in the high denitrification rate and NOx-N removal efficiencies of the acetate and ethanol-fed DNBFs. However, methylotrophic bacteria (*Methyloversatilis* and *Methylotenera*) exclusively dominated the methanol enhanced DNBF.

## Additional file

10.1186/s40064-016-3451-3 T-RFLP fingerprints of biofilm samples from different external carbon source fed DNBFs. R1, R2 and R3 were acetate, ethanol and methanol-fed DNBF. BS1, BS2 and BS3 were biofilm sampling at 0, 200 and 400 mm from the bottom of the filter layer. CB and AB were captured biofilm and attached biofilm. **Figure S2.** Venn diagram of the OTUs for the acetate, ethanol and methanol-fed biofilm. **Table S1.** OUTs on T-RFLP profile combined clone libraries and silico enzymatic digestion.
