# Supplementary material for: Microbial diversity and community structure of denitrifying biological filters operated with different carbon sources
Source: Springerplus. 2016 Oct 7;5(1):1752. doi: 10.1186/s40064-016-3451-3 (PMC5055511; doi:10.1186/s40064-016-3451-3)
Supplement: Supplementary file 1 — 10.1186/s40064-016-3451-3 T-RFLP fingerprints of biofilm samples from different external carbon source fed DNBFs. R1, R2 and R3 were acetate, ethanol and methanol-fed DNBF. BS1, BS2 and BS3 were biofilm sampling at 0, 200 and 400 mm from the bottom of the filter layer. CB and AB were captured biofilm and attached biofilm. Figure S2. Venn diagram of the OTUs for the acetate, ethanol and methanol-fed biofilm. Table S1. OUTs on T-RFLP profile combined clone libraries and silico enzymatic digestion. [file 40064_2016_3451_MOESM1_ESM.docx]

**Supplementary material**

**Fig. S1**. T-RFLP fingerprints of biofilm samples from different external carbon source fed DNBFs. R1, R2 and R3 were acetate, ethanol and methanol-fed DNBF. BS1, BS2 and BS3 were biofilm sampling at 0, 200 and 400 mm from the bottom of the filter layer. CB and AB were captured biofilm and attached biofilm.

**Fig. S2.** Venn diagram of the OTUs for the acetate, ethanol and methanol-fed biofilm.

**Table S1** OUTs on T-RFLP profile combined clone libraries and silico enzymatic digestion

**Fig. S1**. T-RFLP fingerprints of biofilm samples from different external carbon source fed DNBFs. R1, R2 and R3 were acetate, ethanol and methanol-fed DNBF. BS1, BS2 and BS3 were biofilm sampling at 0, 200 and 400 mm from the bottom of the filter layer. CB and AB were captured biofilm and attached biofilm.

|  |
| --- |
|  |
|  |
|  |
|   |
|    |

**Fig. S2.** Venn diagram of the OTUs for the acetate, ethanol and methanol-fed biofilm.

**Table S1**

OUTs on T-RFLP profile combined clone libraries and silico enzymatic digestion

| No | OUT | T-RF(bp) | R1 | | R2 | | R3 | |
| --- | --- | --- | --- | --- | --- | --- | --- | --- |
|  |  |  | Clones | Abundance (%) | Clones | Abundance (%) | Clones | Abundance (%) |
| 1 | OUT 1 | 78 |  |  |  |  | 2 | 10.0 |
| 2 | OUT 2a | 79 | 26 | 66.7 | 26 | 32.5 |  |  |
| 3 | OUT 2b | 79 | 1 | 2.6 | 12 | 15 |  |  |
| 4 | OUT 3 | 82 |  |  | 1 | 1.25 |  |  |
| 5 | OUT 4 | 87 | 2 | 5.1 |  |  |  |  |
| 6 | OUT 5 | 95 |  |  | 1 | 1.25 |  |  |
| 7 | OUT 6 | 121 | 1 | 2.6 |  |  |  |  |
| 8 | OUT 7 | 129 |  |  | 1 | 1.25 |  |  |
| 9 | OUT 8a | 161 |  |  | 1 | 1.25 |  |  |
| 10 | OUT 8b | 161 |  |  | 8 | 10.0 |  |  |
| 11 | OUT 9 | 192 |  |  |  |  | 1 | 5.0 |
| 12 | OUT 10a | 205 |  |  | 1 | 1.25 |  |  |
| 13 | OUT 10b | 205 |  |  | 2 | 2.5 |  |  |
| 14 | OUT 11 | 423 | 1 | 2.6 |  |  |  |  |
| 15 | OUT 12a | 430 | 1 | 2.6 | 26 | 32.5 |  |  |
| 16 | OUT 12b | 430 | 1 | 2.6 |  |  |  |  |
| 17 | OUT 12c | 430 | 3 | 7.7 |  |  |  |  |
| 18 | OUT 12d | 430 |  |  | 1 | 1.25 |  |  |
| 19 | OUT 13 | 437 |  |  |  |  | 1 | 5.0 |
| 20 | OUT 14 | 464 |  |  |  |  | 1 | 5.0 |
| 21 | OUT 15 | 489 | 1 | 2.6 |  |  |  |  |
| 22 | OUT 16a | 490 | 1 | 2.6 |  |  |  |  |
| 23 | OUT 16b | 490 |  |  |  |  | 11 | 55.0 |
| 24 | OUT 16c | 490 |  |  |  |  | 4 | 20.0 |
| 25 | OUT 17 | 511 | 1 | 2.6 |  |  |  |  |
